# Enhanced surface plasmon resonance biosensor with graphene-black phosphorus heterostructure for ultra-high sensitivity refractive index detection with machine learning for behaviour prediction

**DOI:** 10.1371/journal.pone.0332356

**Published:** 2025-11-07

**Authors:** Jacob Wekalao, Hussein A. Elsayed, Ahmed Mehaney, Amuthakkannan Rajakannu, Haifa A. Alqhtani, May Bin-Jumah, Jonas Muheki, Stefano Bellucci

**Affiliations:** 1 Department of Optics and Optical Engineering, University of Science and Technology of China, 96 Jinzhai Road, Hefei, China; 2 Department of Physics, College of Science, University of Ha’il, Ha’il, Saudi Arabia; 3 Photonic and Phononic Crystals Lab., Physics Department, Faculty of Science, Beni-Suef University, Beni-Suef, Egypt; 4 Department Of Mechanical And Industrial Engineering, College Of Engineering, National University Of Science And Technology, Sultanate Of Oman, Oman, Muscat; 5 Department of Biology, College of Science, Princess Nourah bint Abdulrahman University, Riyadh, Saudi Arabia; 6 Department of Physics, University of Houston, Houston, Texas, United States of America; 7 INFN-Laboratori Nazionali di Frascati, Via E. Fermi 54, Frascati, Italy; MNNIT Allahabad: Motilal Nehru National Institute of Technology, INDIA

## Abstract

This study reports a five-layer surface plasmon resonance biosensor architecture comprising a BK7 glass substrate, silver plasmonic film, monolayer graphene, black phosphorus dielectric, and analyte region, engineered for high-precision detection of low refractive index media. The graphene–black phosphorus heterostructure synergistically exploits the exceptionally high surface-to-volume ratio of graphene and the anisotropic optical response of black phosphorus, enabling pronounced electromagnetic field confinement at the sensor interface. In particular, the detection procedure is mainly dependent on the emergence of the angular surface plasmon resonance based on the optimum values of the different geometrical and structural parameters. Therefore, the electromagnetic optimization using COMSOL Multiphysics was performed by varying the silver thickness, graphene thickness and black phosphorus thickness over an analyte index range of 1.29–1.38 RIU. The optimized configuration achieved a maximum sensitivity of 300°/RIU at n = 1.35 RIU, with a figure of merit of 45.455 RIU^–1^ and a detection limit of 0.018 RIU, surpassing previously reported architectures. Furthermore, predictive validation employing K-nearest neighbours regression demonstrated excellent reliability, yielding R² values between 92–100% and mean absolute errors of 0.005–0.012 RIU.

## Introduction

Surface plasmon resonance (SPR) biosensing technology has fundamentally transformed analytical biochemistry and diagnostic applications since its initial development in the 1980s [1,2]. This label-free detection methodology exploits the electromagnetic phenomenon occurring at noble metal-dielectric interfaces, where incident polarized light excites collective electron oscillations known as surface plasmons [3,4]. The characteristic angular dependence of this optical coupling manifests as a pronounced reflectance minimum, whose position correlates directly with refractive index variations in the immediate vicinity of the metallic surface [5,6]. Consequently, biomolecular recognition events produce measurable shifts in the plasmon resonance condition, enabling quantitative analysis without exogenous labelling requirements [7].

Meanwhile, the fundamental operating principle relies on attenuated total reflection through high-refractive-index optical elements, typically prisms, which facilitate efficient photon-plasmon coupling [8,9]. This configuration has evolved from laboratory-scale instrumentation to miniaturized point-of-care devices, though traditional implementations exhibit limitations in detecting low-molecular-weight species and narrow operational ranges [10,11]. The inherent advantages of SPR methodology include preservation of native biomolecular conformations, real-time kinetic analysis capabilities, exceptional molecular specificity through appropriate surface functionalization, minimal sample volume requirements, and compatibility with complex biological matrices [12–14].

Recently, the integration of graphene, a two-dimensional carbon allotrope with sp²-hybridized atomic structure, has revolutionized SPR biosensor performance by addressing sensitivity constraints inherent to conventional metallic substrates [15,16]. Notably, graphene’s exceptional surface area-to-volume ratio (~2630 m²/g) provides extensive probe immobilization capacity, besides its unique optical properties that could provide some improvements on the response of the electromagnetic field at the sensor interface [17,18]. When deposited on gold surfaces, graphene functions as a dielectric spacer that amplifies local field intensities, significantly improving detection sensitivity for small molecular targets that typically produce minimal refractive index perturbations [19,20]. The material’s inherent biocompatibility, chemical stability, and versatile functionalization chemistry through both covalent and non-covalent mechanisms further enhance its utility in biosensing applications [21–23].

Beyond graphene, alternative two-dimensional materials offer complementary advantages for SPR enhancement [24,25]. Transition metal dichalcogenides, including molybdenum disulfide and tungsten diselenide, possess semiconducting properties with direct bandgaps enabling photoluminescent modulation of plasmonic signals through exciton-plasmon coupling mechanisms [26–28]. Hexagonal boron nitride serves as an atomically smooth, chemically inert protective layer that prevents metallic oxidation while maintaining thermal stability [29–31]. MXenes, characterized by high electrical conductivity and abundant surface functional groups, enable dual-modal optical-electrical detection schemes with demonstrated femtomolar sensitivity for cancer biomarker detection [32,33].

In the recent years, some of the advances in biosensing include: S et al. reported that SPR sensors demonstrate reliability with accuracy values ranging from 0.75 to 0.95, alongside notably high sensitivity and specificity [34]. Uniyal et al. reported that the InP–Ti₃C₂Tx MXene SPR sensor achieves a maximum sensitivity of 263.57°/RIU, with a detection accuracy of 0.207/ ° and a figure of merit of 34.62 RIU^–1^ over an RI range of 1.33–1.40 [35]. Ahmed et al. highlighted that SPR enables highly sensitive, label-free detection of molecular interactions, with MXenes offering enhanced conductivity and surface area to further improve sensor performance [36]. Kumar et al. reported that the SPR sensor achieved maximum wavelength sensitivities of 5350.87 nm/RIU (RNA), 5333.33 nm/RIU (spike RBD), and 4700.85 nm/RIU (IgG), with limits of detection of 1.86 × 10 ⁻ ⁶, 2.14 × 10 ⁻ ⁶, and 2.12 × 10 ⁻ ⁶ RIU, respectively, and a penetration depth of 218.07 nm [37]. Shivangani et al. reported that the perovskite–silver SPR sensor achieves a maximum sensitivity of 410.8°/RIU, a detection accuracy of 0.21 ° ⁻ ¹, and a quality factor of 105.18 RIU ⁻ ¹ for early-stage malaria detection [38].Tiwari et al. reported that their Attentive Spec ExLSTM model achieved robust performance for Au-TFBG sensor data quality enhancement, with RMSE = 1.73 ± 0.05, MAE = 1.20 ± 0.04, SMAPE = 2.22 ± 0.05, and a novel minima difference metric of 1.08 ± 0.46. [39]. Rana et al. demonstrated that Cat Boost ML integrated with XAI effectively predicted the FoM of an SPR-based FOSD across 32,768 data points, with SHAP analysis revealing analyte RI and wavelength as the most influential factors governing sensor performance [40]. Srivastava et al. applied an inverse design approach using PSO and TMM to optimize SPR sensor structures, achieving a high sensitivity of 630.54°/RIU and FoM of 2277 RIU^–1^, significantly surpassing previously reported designs [41].

Despite advances in graphene-based and alternative 2D material SPR sensors, most reported designs either focus on high-index analytes or lack machine learning-based predictive modelling. Furthermore, black phosphorus has rarely been combined with graphene to target low refractive index analytes, despite its anisotropic optical properties. This work addresses this gap by introducing a graphene–BP heterostructure SPR sensor, optimized for low-RI detection and validated with KNN regression, thus bridging material innovation with predictive intelligence.

### Design and modelling

**Fig 1:**
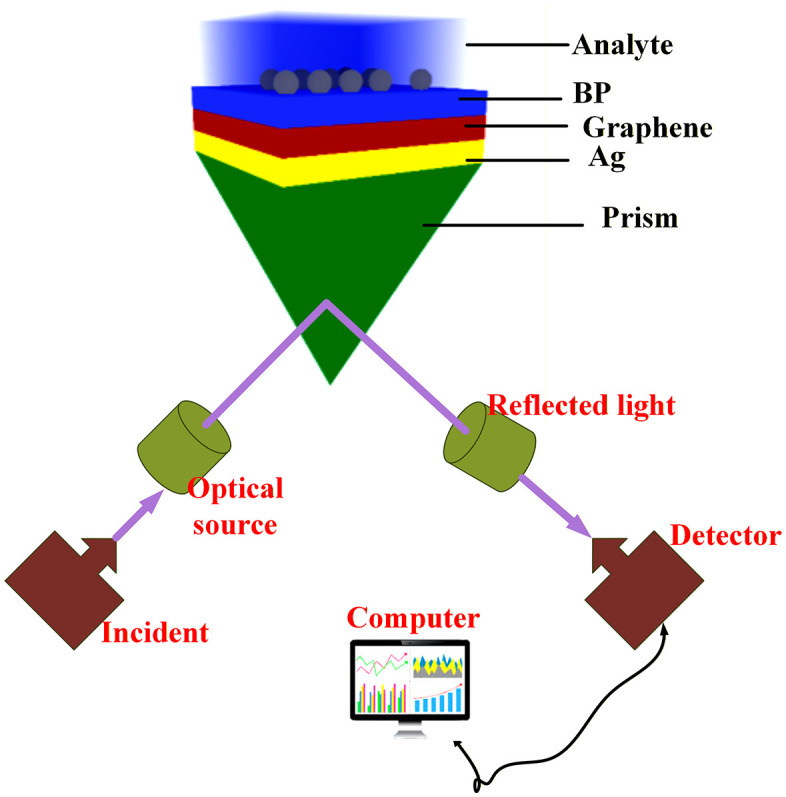
Schematic representation of the five-layer SPR sensor architecture showing BK7 prism, silver film, graphene monolayer, black phosphorus layer, and analyte medium with their respective thicknesses.

This work presents a five-layer surface plasmon resonance (SPR) sensing platform featuring a hierarchical architecture. The device comprises a BK7 glass substrate serving as the optical coupling element, followed by a silver (Ag) plasmonic film, a single-layer graphene sheet, a black phosphorus dielectric component, and the target analyte region. The silver plasmonic layer is engineered with a 40–65 nm thickness to optimize surface plasmon wave generation at the metal-dielectric boundary. The graphene maintains its characteristic 3–6nm nm thickness, providing exceptional electronic properties. The black phosphorus layer is precisely controlled at 1–8.2nm nm thickness to maximize optical field enhancement and strengthen plasmonic confinement effects. For sensing validation, two distinct analyte refractive index values (1.29 and 1.38) are selected to simulate practical conditions encountered in biochemical detection applications. These values represent typical RI variations found in real-world sensing scenarios. The wavelength-dependent optical properties of silver, graphene, and black phosphorus are calculated using Sellmeier equation parameters, ensuring accurate representation of their dispersive behaviour across the operational spectrum. The BK7 substrate facilitates proper momentum matching requirements for plasmon coupling, while the graphene-black phosphorus heterostructure provides superior field localization, elevated sensitivity response, and reduced ohmic losses compared to conventional metallic configurations. Electromagnetic simulations are performed using COMSOL Multiphysics 6.2 software, implementing finite element methodology to evaluate the reflectance response of the multilayer sensor architecture. Sensitivity characterization is achieved by monitoring spectral shifts in the reflectance minima as a function of analyte refractive index variations. The sensor geometry is depicted schematically in **Fig 1**, showing the layer sequence and dimensional relationships.

The novelty of this work lies in three aspects: (i) the synergistic use of graphene and black phosphorus, whose combined anisotropic and plasmonic properties yield superior field localization and sensitivity; (ii) optimization of sensor design specifically for low refractive index detection (1.29–1.38 RIU), a regime critical for biological and environmental samples; and (iii) the integration of K-nearest neighbors regression, which ensures predictive robustness and real-world adaptability. These advances collectively position our sensor as a transformative platform in SPR biosensing.

**Fig 2:**
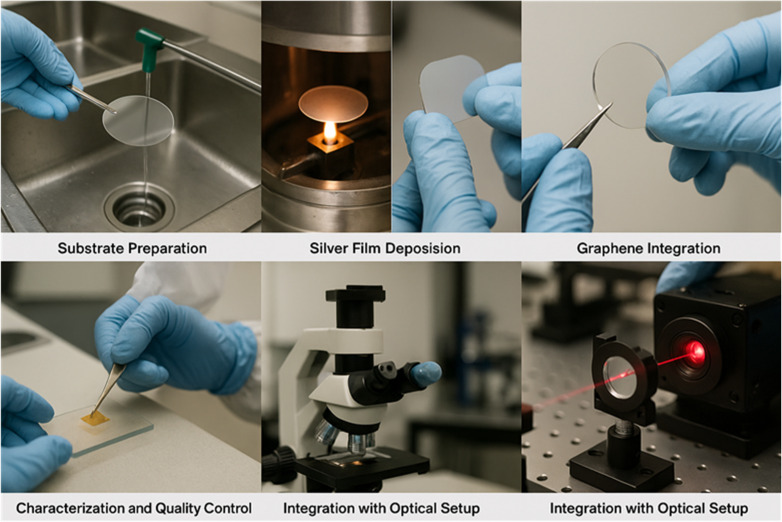
Step-by-step fabrication and integration process of the proposed five-layer SPR sensor, including substrate preparation, silver film deposition, graphene integration, black phosphorus layer fabrication, characterization, and final optical setup assembly.

In this regard, the fabrication of the five-layer SPR sensor as demonstrated in Fig 2 begins with substrate preparation, where BK7 glass wafers are rigorously cleaned to achieve atomically smooth surfaces. A thin silver plasmonic layer is then deposited using electron-beam evaporation or sputtering under ultra-high vacuum, often preceded by a chromium or titanium adhesion layer. This is followed by the delicate transfer of a monolayer graphene sheet, grown via chemical vapour deposition or obtained commercially, onto the silver surface. Residual polymers from the transfer process are removed through annealing to preserve graphene’s electronic quality. The integration of a thin black phosphorus (BP) layer, exfoliated or synthesized under inert conditions, is particularly challenging due to its air sensitivity, requiring immediate encapsulation or protective coatings such as Al₂O₃.After the layered structure is fabricated, engineers employ advanced characterization techniques, including ellipsometry for thickness monitoring, Raman spectroscopy for graphene quality, and atomic force microscopy for surface morphology. Once verified, the device is coupled with a precision optical system using the Kretschmann configuration, enabling surface plasmon resonance excitation with a laser source. Automated angular scanning and lock-in amplification are used for high-resolution signal acquisition. This multi-step process demonstrates the integration of nanomaterials into a functional biosensing platform, balancing theoretical design performance with real-world fabrication challenges. However, there are some real environmental factors that could be effective on the overall performance of the designed SPR sensor. A key challenge in fabricating BP-based devices lies in its air sensitivity, as exposure to oxygen and moisture leads to degradation. To mitigate this, encapsulation methods such as atomic-layer-deposited Al₂O₃ coatings, hexagonal boron nitride (hBN) encapsulation, or polymer over-layers can be employed. These approaches stabilize BP’s structure while preserving its anisotropic optical properties, ensuring reliability for biosensing applications.

### Electromagnetic analysis of five-layer surface plasmon resonance sensor

The electromagnetic field distribution in the multilayered structure is governed by the time-harmonic Maxwell equations:


$$\begin{array}{cc} \nabla \times 𝐄 & =-j\omega \mu_{\text{0}}𝐇\\ \nabla \times 𝐇 & =j\omega \varepsilon_{\text{0}}\varepsilon_{r}(𝐫)𝐄+𝐉 \end{array}$$ (1)

where 𝐄 and 𝐇 represent the complex electric and magnetic field vectors, ω is the angular frequency, $\mu_{\text{0}}$ and $\varepsilon_{\text{0}}$ are the free-space permeability and permittivity, respectively and $\varepsilon_{r}(𝐫)$ denotes the position-dependent relative permittivity tensor.

For a stratified geometry with interfaces parallel to the xy-plane, the electromagnetic fields decompose into transverse electric (TE) and transverse magnetic (TM) polarization modes. The TM polarization enables SPR excitation through normal electric field coupling with surface plasmons. Notably, in the case of TM polarization, the propagation constant of the surface plasmon waves is equivalent to that of the propagating electromagnetic waves which in turns leads to the emergence of the SPR mode.

In this context, the electromagnetic field continuity across each interface is enforced using the transfer matrix approach. For each layer j with thickness $d_{j}$, the transfer matrix ${𝐌}_{j}$ relates field amplitudes at boundaries:


$$\left[\begin{array}{c} E_{+}^{j+\text{1}}\\ E_{-}^{j+\text{1}} \end{array}]=[\begin{array}{cc} M_{\text{1}\text{1}}^{j} & M_{\text{1}\text{2}}^{j}\\ M_{\text{2}\text{1}}^{j} & M_{\text{2}\text{2}}^{j} \end{array}][\begin{array}{c} E_{+}^{j}\\ E_{-}^{j} \end{array}\right]$$ (2)

where $E_{+}^{j}$ and $E_{-}^{j}$ represent forward and backward propagating wave amplitudes in layer j. For TM polarization mode, the transfer matrix elements are:


$$\begin{array}{cc} M_{\text{1}\text{1}}^{j} & =\text{cos}(k_{z}^{j}d_{j})\\ M_{\text{1}\text{2}}^{j} & =\frac{jk_{z}^{j}}{\varepsilon_{r}^{j}}\text{sin}(k_{z}^{j}d_{j})\\ M_{\text{2}\text{1}}^{j} & =\frac{j\varepsilon_{r}^{j}}{k_{z}^{j}}\text{sin}(k_{z}^{j}d_{j})\\ M_{\text{2}\text{2}}^{j} & =\text{cos}(k_{z}^{j}d_{j}) \end{array}$$ (3)

where $k_{z}^{j}=\sqrt{\varepsilon_{r}^{j}k_{\text{0}}^{\text{2}}-k_{x}^{\text{2}}}$ is the z-component of the wave vector in layer j, $k_{\text{0}}=\omega /c$ is the free-space wave number, and $k_{x}$ is the conserved tangential wave vector component.

Then, silver’s permittivity follows the extended Drude-Lorentz model incorporating interband transitions:


$$\varepsilon_{\text{Ag}}(\omega )=\varepsilon_{\infty }-\frac{\omega_{p}^{\text{2}}}{\omega^{\text{2}}+j\gamma_{p}\omega }+\sum_{i}\frac{f_{i}\omega_{i}^{\text{2}}}{\omega_{i}^{\text{2}}-\omega^{\text{2}}-j\gamma_{i}\omega }$$ (4)

where:

- $\varepsilon_{\infty }=\text{5}.\text{0}$ is the high-frequency permittivity
- $\omega_{p}=\text{1}.\text{3}\text{8}\times {\text{1}\text{0}}^{\text{1}\text{6}}$ rad/s is the plasma frequency
- $\gamma_{p}=\text{2}.\text{7}\text{3}\times {\text{1}\text{0}}^{\text{1}\text{3}}$ rad/s is the damping coefficient
- The summation accounts for interband contributions with oscillator strengths $f_{i}$, resonance frequencies $\omega_{i}$, and damping parameters $\gamma_{i}$

Then, graphene’s conductivity is modeled using the Kubo formula, accounting for both intraband and interband contributions:


$$\sigma_{g}(\omega ,\mu_{c},\Gamma ,T)=\sigma_{\text{intra}}(\omega ,\mu_{c},\Gamma ,T)+\sigma_{\text{inter}}(\omega ,\mu_{c},T)$$ (5)

The intraband conductivity is:


$$\sigma_{\text{intra}}=\frac{je^{\text{2}}k_{B}T}{\pi \hbar^{\text{2}}(\omega +j\Gamma )}\text{ln}\left[\text{2}\text{cosh}\left(\frac{\mu_{c}}{\text{2}k_{B}T}\right)\right]$$ (6)

The interband conductivity involves:


$$\sigma_{\text{inter}}=\frac{je^{\text{2}}}{\text{4}\pi \hbar }\left[G\left(\frac{\hbar \omega }{\text{2}}\right)+j\frac{\text{4}\hbar \omega }{\pi }\int_{\text{0}}^{\infty }\frac{G(\xi )-G(\hbar \omega /\text{2})}{(\hbar \omega {)}^{\text{2}}-\text{4}\xi^{\text{2}}}d\xi \right]$$ (7)

where:


$$G(\xi )=\frac{\text{sinh}(\xi /k_{B}T)}{\text{cosh}(\mu_{c}/k_{B}T)+\text{cosh}(\xi /k_{B}T)}$$ (8)

with $\mu_{c}$ as chemical potential, Γ as scattering rate, T as temperature, and $k_{B}$ as Boltzmann’s constant.

The effective graphene permittivity is:


$$\varepsilon_{g}=\text{1}+\frac{j\sigma_{g}}{\varepsilon_{\text{0}}\omega t_{g}}$$ (9)

where $t_{g}=\text{0}.\text{3}\text{3}\text{5}$ nm is the graphene monolayer thickness.

Black phosphorus exhibits strong in-plane anisotropy with the permittivity tensor:


$$\varepsilon_{\text{BP}}=[\begin{array}{ccc} \varepsilon_{xx} & \text{0} & \text{0}\\ \text{0} & \varepsilon_{yy} & \text{0}\\ \text{0} & \text{0} & \varepsilon_{zz} \end{array}]$$ (10)

The diagonal components follow wavelength-dependent Sellmeier relationships:


$$\begin{array}{cc} \varepsilon_{xx}(\lambda ) & =A_{\text{1}}+\frac{B_{\text{1}}\lambda^{\text{2}}}{\lambda^{\text{2}}-C_{\text{1}}^{\text{2}}}+\frac{D_{\text{1}}\lambda^{\text{2}}}{\lambda^{\text{2}}-E_{\text{1}}^{\text{2}}}\\ \varepsilon_{yy}(\lambda ) & =A_{\text{2}}+\frac{B_{\text{2}}\lambda^{\text{2}}}{\lambda^{\text{2}}-C_{\text{2}}^{\text{2}}}+\frac{D_{\text{2}}\lambda^{\text{2}}}{\lambda^{\text{2}}-E_{\text{2}}^{\text{2}}} \end{array}$$ (11)

where $\left(A_{\text{1}},B_{\text{1}},C_{\text{1}},D_{\text{1}},E_{\text{1}}\right)$ and $\left(A_{\text{2}},B_{\text{2}},C_{\text{2}},D_{\text{2}},E_{\text{2}}\right)$ are experimentally determined Sellmeier parameters specific to crystallographic orientations.

The surface plasmon polariton (SPP) dispersion relation at the metal-dielectric interface is derived from boundary conditions requiring continuous tangential electric and magnetic fields:


$$k_{\text{SPP}}=k_{\text{0}}\sqrt{\frac{\varepsilon_{m}\varepsilon_{d}}{\varepsilon_{m}+\varepsilon_{d}}}$$ (12)

where $\varepsilon_{m}$ and $\varepsilon_{d}$ are the complex permittivities of metal and dielectric layers.

For the multilayer configuration, the modified dispersion relation becomes:


$$\text{det}\left|M_{\text{1}\text{1}}^{\text{total}}+M_{\text{2}\text{2}}^{\text{total}}\right|=\text{0}$$ (13)

where ${𝐌}^{\text{total}}={𝐌}_{\text{BK7}}\times {𝐌}_{\text{Ag}}\times {𝐌}_{\text{graphene}}\times {𝐌}_{\text{BP}}\times {𝐌}_{\text{analyte}}$ is the total system transfer matrix.

The electric field enhancement factor is calculated as:


$$\eta (z)=\frac{|E_{z}(z){|}^{\text{2}}}{|E_{\text{0}}{|}^{\text{2}}}$$ (14)

where $E_{z}(z)$ is the z-component of the electric field at position z, and $E_{\text{0}}$ is the incident field amplitude.

The field confinement length is determined by:


$$L_{\text{conf}}=\frac{\int_{-\infty }^{\infty }|E_{z}(z){|}^{\text{2}}dz}{\text{max}\{|E_{z}(z){|}^{\text{2}}\}}$$ (15)

Now, to assess the overall performance of the designed SPR sensor, we have to define some parameters including sensitivity and figure of merit. The bulk sensitivity is defined as the spectral shift per unit refractive index change:


$$S_{B}=\frac{\Delta \lambda_{\text{res}}}{\Delta n}=\frac{\partial \lambda_{\text{res}}}{\partial n}$$ (16)

where $\lambda_{\text{res}}$ is the resonance wavelength and n is the analyte refractive index. The figure of merit (FOM) incorporates both sensitivity and spectral resolution:


$$\text{FOM}=\frac{S_{B}}{\text{FWHM}}$$ (17)

Finally, the reflectance coefficient for the complete multilayer system is:


$$R=|r_{\text{1}\text{2}}^{\text{total}}{|}^{\text{2}}={\left|\frac{M_{\text{2}\text{1}}^{\text{total}}}{M_{\text{1}\text{1}}^{\text{total}}}\right|}^{\text{2}}$$ (18)

The phase change upon reflection is:


$$\Phi =\text{arg}(r_{\text{1}\text{2}}^{\text{total}})=\text{arg}\left(\frac{M_{\text{2}\text{1}}^{\text{total}}}{M_{\text{1}\text{1}}^{\text{total}}}\right)$$ (19)

The analysis considers few-layer graphene (1–6 nm), as experimentally realized devices often exceed a single atomic layer. Few-layer graphene enhances plasmon confinement by increasing carrier density, but beyond ~5–6 nm, optical absorption losses can offset these benefits, necessitating thickness optimization.

## Results and discussion

The graphene-based refractive index (RI) sensor illustrated in Fig 1 was numerically simulated using COMSOL Multiphysics, and the corresponding reflectance results are presented in this section. In COMSOL simulations, perfectly matched layer (PML) boundaries were applied to minimize reflections at the edges. A physics-controlled mesh with refinement up to λ/15 was used around graphene and BP interfaces to capture near-field effects accurately. The iterative solver was run until convergence was achieved with a relative error tolerance of 1 × 10 ⁻ ⁶ in reflectance values, ensuring reproducibility and numerical stability*.* The sensor optimization involved systematically varying key parameters, including the analyte refractive index $n_{\text{analyte}}$, the silver (Ag) layer thickness $d_{\text{Ag}}$, and the angle of incidence θ of the excitation beam, which was swept from ${\text{6}\text{5}}^{\circ }$ to ${\text{9}\text{0}}^{\circ }$. The analyte RI was varied over the range 1.29–1.38, while the Ag thickness was adjusted from 40 nm to 65 nm in increments of 5 nm to investigate their combined effect on reflectance. The reflectance R of the prism–metal–analyte multilayer system can be modeled using extended Fresnel relations:


$$R={\left|\frac{r_{\text{0}\text{1}}+r_{\text{1}\text{2}}\;e^{\text{2}ik_{\text{1}}d_{\text{Ag}}\text{cos}\theta }}{\text{1}+r_{\text{0}\text{1}}r_{\text{1}\text{2}}\;e^{\text{2}ik_{\text{1}}d_{\text{Ag}}\text{cos}\theta }}\right|}^{\text{2}},$$ (20)

where the Fresnel coefficients at the prism/Ag and Ag/analyte interfaces are:


$$r_{\text{0}\text{1}}=\frac{n_{\text{0}}\text{cos}\theta -\sqrt{n_{\text{Ag}}^{\text{2}}-n_{\text{0}}^{\text{2}}{\text{sin}}^{\text{2}}\theta }}{n_{\text{0}}\text{cos}\theta +\sqrt{n_{\text{Ag}}^{\text{2}}-n_{\text{0}}^{\text{2}}{\text{sin}}^{\text{2}}\theta }},\;r_{\text{1}\text{2}}=\frac{\sqrt{n_{\text{Ag}}^{\text{2}}-n_{\text{0}}^{\text{2}}{\text{sin}}^{\text{2}}\theta }-n_{\text{analyte}}\text{cos}\theta_{\text{2}}}{\sqrt{n_{\text{Ag}}^{\text{2}}-n_{\text{0}}^{\text{2}}{\text{sin}}^{\text{2}}\theta }+n_{\text{analyte}}\text{cos}\theta_{\text{2}}},$$ (21)

with the propagation constant in the Ag layer defined as


$$k_{\text{1}}=\frac{\text{2}\pi n_{\text{Ag}}}{\lambda },\;\text{cos}\theta_{\text{2}}=\sqrt{\text{1}-{\left(\frac{n_{\text{0}}}{n_{\text{analyte}}}\text{sin}\theta \right)}^{\text{2}}}.$$ (22)

In this study, the sensor sensitivity is defined as the change in the resonance angle per unit change in analyte refractive index:


$$S=\frac{d\theta }{dn_{\text{analyte}}}.$$ (23)

**Fig 3:**
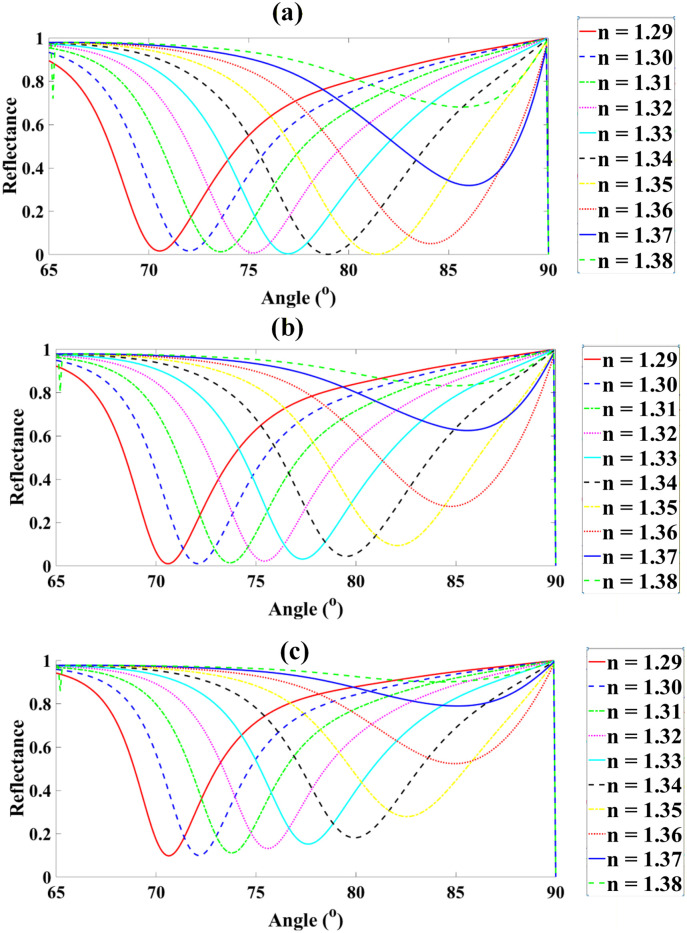
Reflectance spectra of the graphene-based sensor for varying Ag thicknesses at analyte RIs from 1.29 to 1.38.

The reflectance spectra demonstrate a strong dependence on both Ag layer thickness and analyte RI. For the Ag thickness of 40 nm, the reflectance values for $n_{\text{analyte}}$ = 1.29, 1.30, 1.31, 1.32, 1.33, 1.34, 1.35, 1.36, 1.37, and 1.38 are 1.649%, 1.636%, 1.255%, 0.726%, 0.352%, 0.109%, 0.147%, 5.038%, 31.880%, and 68.052%, respectively. Increasing the thickness to 45 nm produces reflectance values of 0.958%, 0.987%, 1.384%, 2.186%, 3.117%, 4.407%, 9.373%, 27.431%, 62.468%, and 80.183%. For 50 nm, the reflectance values are 9.786%, 9.948%, 11.110%, 13.131%, 15.180%, 18.104%, 27.880%, 52.333%, 78.918%, and 86.001%. At 55 nm, the reflectance ranges from 24.822%, 25.066%, 26.756%, 29.390%, 31.964%, 35.819%, 47.594%, 70.270%, 87.419%, to 89.991%. For 60 nm, the corresponding reflectance values are 41.673%, 42.005%, 43.730%, 46.395%, 48.917%, 52.818%, 63.859%, 81.430%, 92.033%, and 92.648%. Finally, the maximum thickness of 65 nm yields reflectance values of 57.052%, 57.384%, 58.911%, 61.204%, 63.324%, 66.701%, 75.675%, 88.151%, 94.641%, and 94.382% across the same analyte RI range. These results indicate that increasing the Ag thickness systematically enhances the reflectance, particularly for higher analyte refractive indices. Furthermore, sweeping the incidence angle from ${\text{6}\text{5}}^{\circ }$ to ${\text{9}\text{0}}^{\circ }$ significantly influences the resonance condition, increasing the coupling of incident light to surface plasmons and leading to sharper reflectance peaks. The defined sensitivity, $S=d\theta /dn_{\text{analyte}}$, provides a quantitative measure of how the resonance angle shifts in response to changes in analyte refractive index, which is essential for high-precision refractive index sensing. The reflectance trends observed with increasing Ag thickness indicate enhanced plasmon coupling efficiency up to an optimal point, beyond which increased damping reduces sensitivity. Similarly, as graphene thickness rises, field confinement intensifies due to improved charge carrier density, though excessive thickness introduces absorption losses. For BP, thicker layers amplify anisotropic field enhancement, but beyond ~5 nm, saturation effects reduce marginal gains. These interpretations confirm the importance of balancing field confinement with material losses in optimizing sensor design as demonstrated in Fig 3.

**Fig 4:**
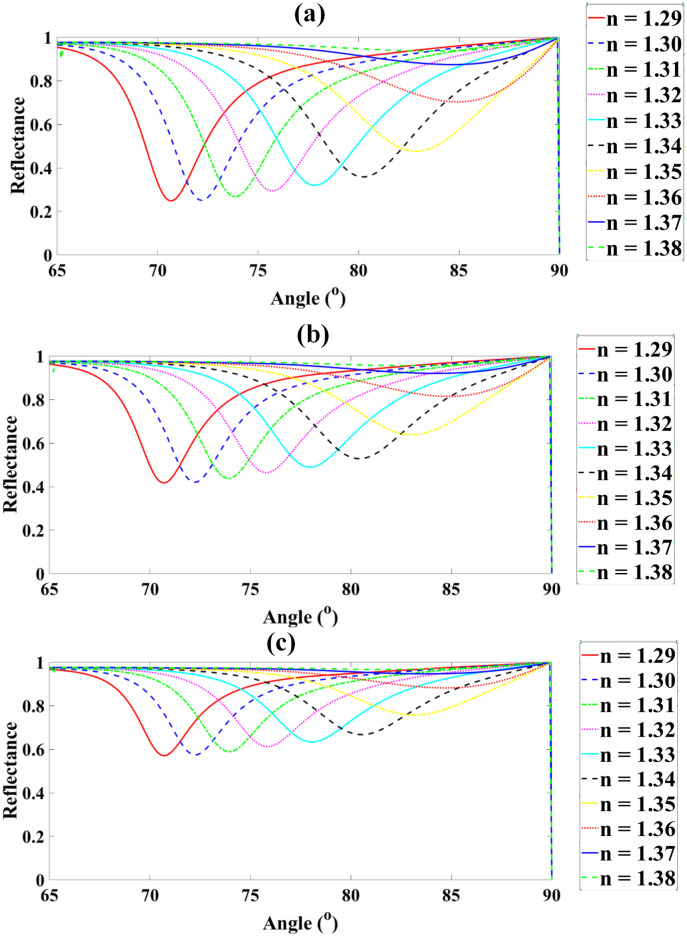
Reflectance spectra of the proposed sensor for analyte RI values from 1.29 to 1.38, showing variation with graphene thickness from 1 nm to 6 nm.

**Fig 5:**
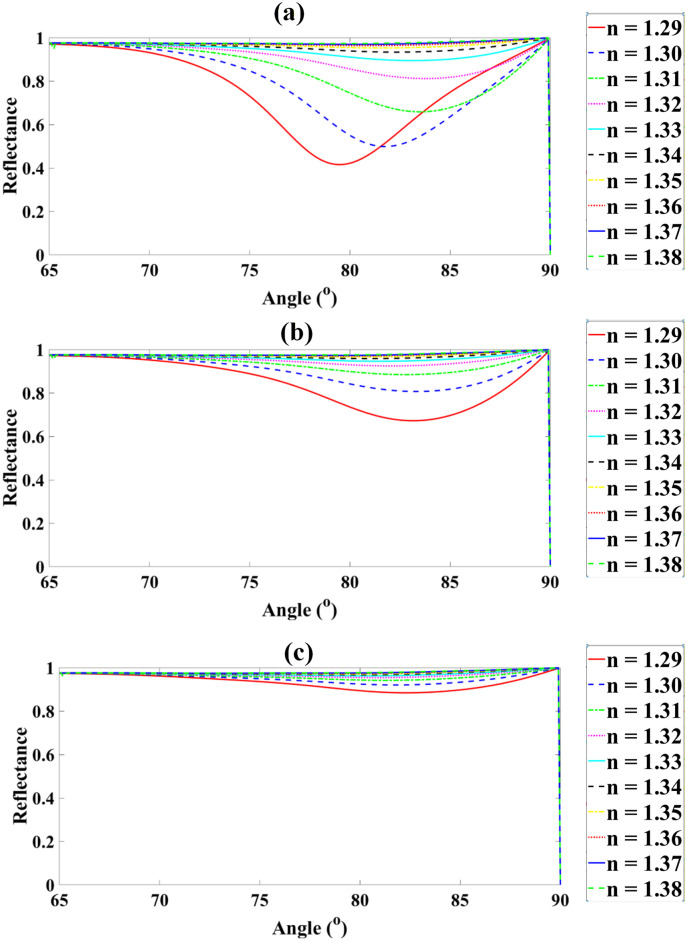
Dependence of peak reflectance on graphene thickness for different analyte RIs, illustrating enhanced sensor response at higher thicknesses.

**Fig 6:**
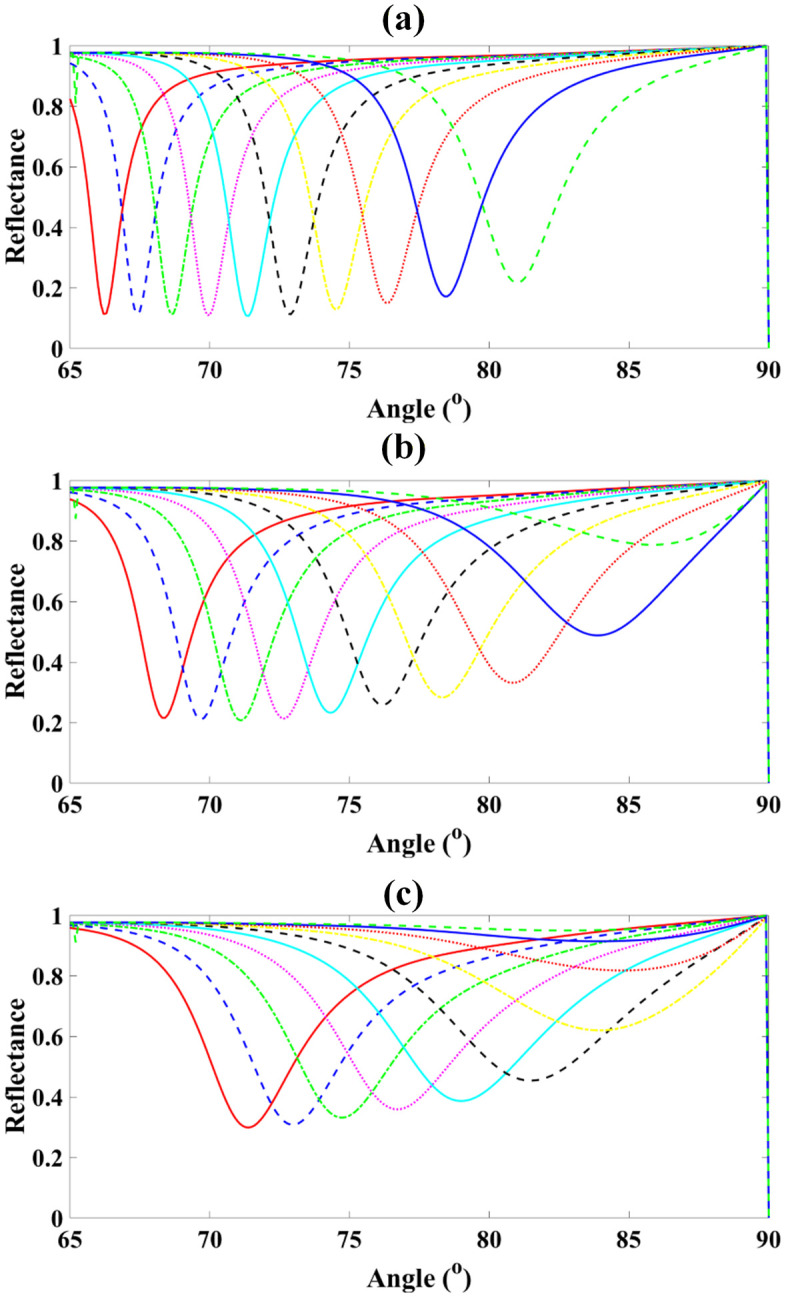
Reflectance spectra of the proposed sensor for RIs 1.29–1.38 with varying Black Phosphorus thicknesses from 1.4 nm to 5.6 nm (steps of 1.2 nm).

**Fig 7:**
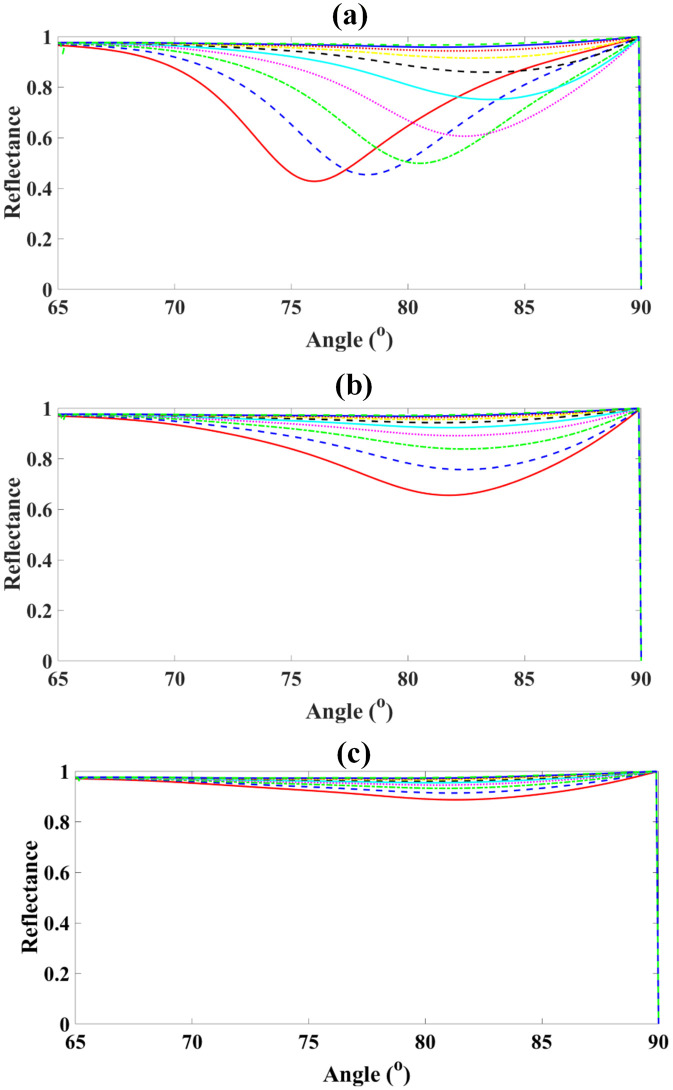
Corresponding reflectance variation as a function of RI for each Black Phosphorus thickness, highlighting enhanced sensitivity at higher thicknesses.

Also as demonstrated in Fig 4a-c, Fig 5a-c, Fig 6a-c and Fig 7a-c,the sensor was evaluated for analyte RI values from 1.29 to 1.38 while varying graphene thickness (1–6 nm) and BP thickness (1.4–5.6 nm). For graphene, reflectance rose progressively from ~27.8% at 1 nm (RI = 1.29) to ~97.4% at 6 nm (RI = 1.37), with near-saturation beyond RI = 1.36. Similarly, for BP, reflectance increased from ~11.3% at 1.4 nm (RI = 1.29) to ~97.2% at 5.6 nm (RI = 1.37). These results demonstrate that increasing both graphene and BP thicknesses enhances plasmonic confinement and reflectance, yielding peak responses above 97% and confirming their critical role in maximizing sensor sensitivity.

**Fig 8:**
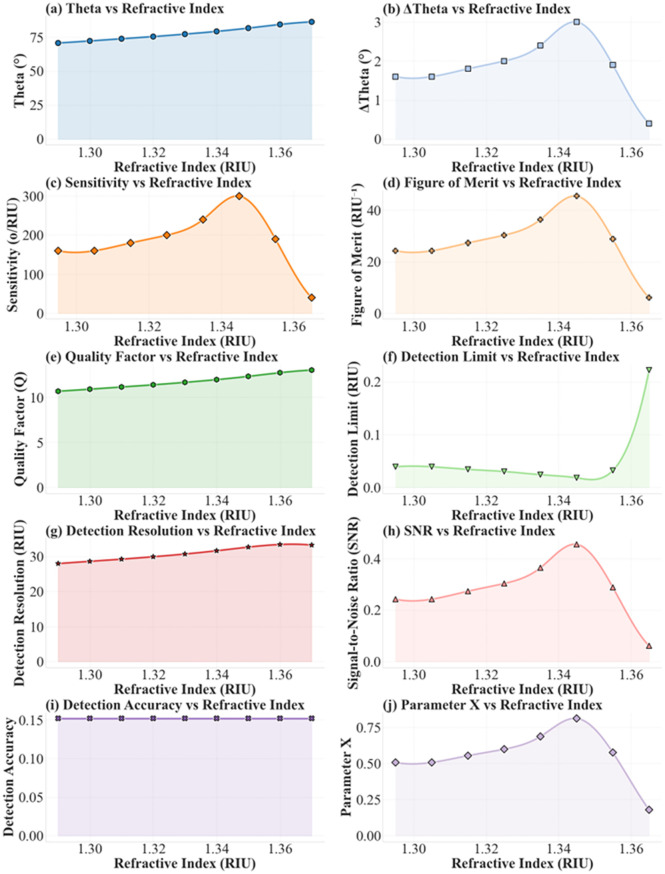
Performance analysis of the proposed sensor as a function of refractive index.

The data in Table 1 and Fig 8 presents the variation of multiple sensor performance parameters with respect to the refractive index (n) ranging from 1.29 to 1.38 RIU. The corresponding resonance angle (θ) increases from 70.5° to a peak of 86° before slightly decreasing to 85.6°. The change in resonance angle (dθ) ranges from 0.4° to 3°, with most intermediate values between 1.5° and 2.4°. The refractive index resolution increment (dn) remains constant at 0.01 RIU across all measurements. Sensitivity (S) shows a rising trend from 150 °/RIU at 1.30 RIU to a maximum of 300 °/RIU at 1.35 RIU, then decreases sharply to 40 °/RIU at 1.38 RIU. The full-width at half-maximum (FWHM) is consistent at 6.6°, leading to figure of merit (FOM) values that increase from 22.727 RIU ⁻ ¹ to 45.455 RIU ⁻ ¹ at 1.35 RIU before dropping to 6.061 RIU ⁻ ¹ at 1.38 RIU. Other key performance indicators also exhibit notable trends. The quality factor (Q) steadily rises from 10.682 to 13.03, while the detection limit (DL) decreases from 0.042 to a minimum of 0.018 at 1.35 RIU, indicating improved detection capability, before sharply increasing to 0.222 at 1.38 RIU. Dynamic range (DR) grows from 28.026 to 33.475, and signal-to-noise ratio (SNR) increases from 0.227 to 0.455 before dropping to 0.061. Sensitivity ratio (SR) decreases from 6.373 to 5.359, then slightly rebounds to 8.868. The differential amplitude (DA) remains constant at 0.152, while the parameter X rises from 0.483 to a peak of 0.812 at 1.35 RIU and then falls to 0.179 at 1.38 RIU. Overall, the numerical trends show that optimal sensor performance is achieved around n = 1.35 RIU, after which most metrics degrade sharply. Despite the sensitivity initially improves with increasing Ag, graphene, and BP thicknesses, the performance plateaus or degrades beyond certain limits. For Ag, excess thickness increases damping losses; for graphene, multilayer absorption reduces field penetration; and for BP, optical field saturation diminishes marginal enhancement. Thus, optimized thickness ranges are critical for maintaining high sensitivity. The RI range of 1.29–1.38 is relevant to practical applications, including protein sensing in biological fluids (1.33–1.37), glucose monitoring in physiological environments (~1.34), and malaria biomarker detection in serum (~1.35). Thus, the proposed sensor targets analytes of direct clinical and biochemical significance.

**Table 1:** Showing the variation of sensor performance parameters with refractive index from 1.29 to 1.38 RIU.

θ (°)	70.5	72	73.6	75.2	77	79	81.4	84.1	86	85.6
n(RIU)	1.29	1.3	1.31	1.32	1.33	1.34	1.35	1.36	1.37	1.38
d θ (°)		1.5	1.6	1.6	1.8	2	2.4	3	1.9	0.4
dn(RIU)		0.01	0.01	0.01	0.01	0.01	0.01	0.01	0.01	0.01
S(^o^/RIU)		150.000	160.000	160.000	180.000	200.000	240.000	300.000	190.000	40.000
FWHM(^o^)	6.600	6.600	6.600	6.600	6.600	6.600	6.600	6.600	6.600	6.600
FOM(RIU^-1^)		22.727	24.242	24.242	27.273	30.303	36.364	45.455	28.788	6.061
Q	10.682	10.909	11.152	11.394	11.667	11.970	12.333	12.742	13.030	12.970
DL		0.042	0.039	0.039	0.034	0.030	0.024	0.018	0.032	0.222
DR		28.026	28.649	29.272	29.972	30.751	31.685	32.736	33.475	33.320
SNR		0.227	0.242	0.242	0.273	0.303	0.364	0.455	0.288	0.061
SR		6.373	6.271	6.271	6.089	5.930	5.666	5.359	6.007	8.868
DA	0.152	0.152	0.152	0.152	0.152	0.152	0.152	0.152	0.152	0.152
X		0.483	0.507	0.507	0.554	0.599	0.687	0.812	0.576	0.179

**Fig 9:**
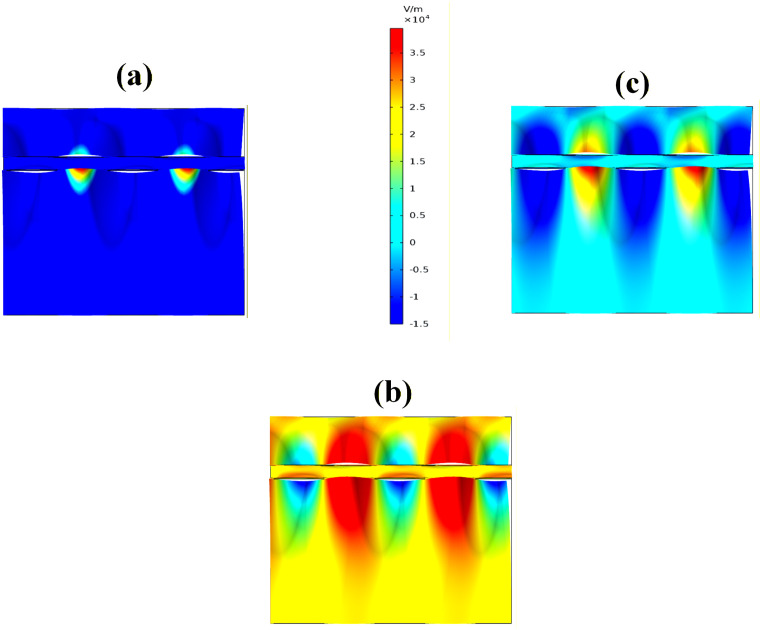
a–c. Electric field distribution of the proposed sensor at 65°, 80°, and 89°, showing maximum reflectance with strong brown field confinement at 80°, and minimum reflectance with dominant blue regions at 65° and 89°.

Then, the electric field distribution results for the proposed sensor design are illustrated in Fig 9a–c for the incidence angles of 65°, 80°, and 89°. At an incidence angle of 80°, the sensor exhibits a strong field confinement, characterized by maximum reflectance and minimum transmission. This effect is clearly visualized by the intense brown coloration localized on the sensing surface, indicating a strong plasmonic resonance and enhanced interaction between the incident light and the active materials. Conversely, at incidence angles of 65° and 89°, the field maps reveal predominantly deep blue coloration across the structure, signifying weaker resonance conditions. This corresponds to minimum reflectance and maximum transmission, which implies reduced sensitivity and weaker coupling efficiency at these angles. The contrast in coloration between the three cases highlights the angular dependence of the sensor’s optical response, with 80° providing the most optimal operating condition due to its superior field enhancement and resonance localization.

### Machine learning

K-nearest neighbours (KNN) regression is a non-parametric machine learning technique that predicts continuous target values by leveraging the principle of local similarity in feature space [42–44]. The algorithm works by identifying the k closest training examples to a query point using a distance metric (typically Euclidean distance), then computing the prediction as a weighted or unweighted average of these neighbours’ target values [45–47]. As an optimization technique, KNN regression is particularly effective for problems where the underlying relationship between features and targets exhibits local patterns or non-linear structures that parametric models might struggle to capture. The method requires careful tuning of the hyperparameter k, where smaller values can lead to overfitting by being too sensitive to noise, while larger values may cause under fitting by over-smoothing local variations. Additionally, feature scaling and distance metric selection are crucial optimization considerations, as KNN’s performance heavily depends on the meaningful measurement of similarity between data points in the feature space [48].

Let $D_{n}=\{(X_{i},Y_{i}{\}}_{i=\text{1}}^{n}$ denote i.i.d. samples drawn from an unknown distribution $P_{X,Y}$ on $(R^{d}\times \text{R},B)$. The regression functional of interest is


$$m^{⋆}(x)=\text{arg}\underset{m(x)\in \text{R}}{\text{min}}E\left[(Y-m(x){)}^{\text{2}}|X=x\right]=\text{E}[Y|X=x],$$ (24)

the Bayes conditional mean.

Define a pseudo-metric space $\left(R^{d},d_{\Omega }\right)$ where the distance is induced by some positive semidefinite form Ω≽0:


$$d_{\Omega }(x,x^{\prime })=((x-x^{\prime }{)}^{⊤}\Omega (x-x^{\prime }){)}^{\text{1}/\text{2}}.$$ (25)

For each query point $x\in R^{d}$, consider the ordered statistics of distances


$$d_{\left(\text{1}\right)}(x)\leq d_{\left(\text{2}\right)}(x)\leq \cdots \leq d_{\left(n\right)}(x),$$ (26)

with associated indices $\pi_{j}(x)$ such that $d_{\left(j\right)}(x)=d_{\Omega }(x,X_{\pi_{j}(x)})$. The corresponding neighbours are $\left(X_{\pi_{j}(x)},Y_{\pi_{j}(x)}\right)$.

### Generalized kNN regressor

The most abstract k-nearest neighbour regressor can be written as


$${\hat{m}}_{k}(x)=\frac{\sum_{j=\text{1}}^{k}\phi \left(\frac{d_{\Omega }\left(x,X_{\pi_{j}(x)}\right)}{h_{k}(x)};\theta \right)\;Y_{\pi_{j}(x)}}{\sum_{j=\text{1}}^{k}\phi \left(\frac{d_{\Omega }\left(x,X_{\pi_{j}(x)}\right)}{h_{k}(x)};\theta \right)},$$ (27)

where $\phi (\cdot ;\theta ):R_{\geq \text{0}}\to R_{\geq \text{0}}$ is a generalized weight-generating functional (e.g., $\phi (r;\theta )=r^{-\alpha }$ or $\text{exp}(-r^{\text{2}}/h^{\text{2}})$), and $h_{k}(x)=d_{\left(k\right)}(x)$ is the adaptive local bandwidth.

An alternative expression is


$${\hat{m}}_{k}(x)=\frac{\sum_{i=\text{1}}^{n}K\left(\frac{d_{\Omega }(x,X_{i})}{h_{k}(x)}\right)Y_{i}}{\sum_{i=\text{1}}^{n}K\left(\frac{d_{\Omega }(x,X_{i})}{h_{k}(x)}\right)},$$ (28)

with kernel K(u)=1{u≤1}. Here the bandwidth is a stochastic quantity depending on the empirical distribution of $\left\{X_{i}\right\}$.

### Bias–variance decomposition

Suppose $Y=m^{⋆}(X)+\varepsilon$, with E[ε∣X]=0 and $\text{Var}(\varepsilon |X)=\sigma^{\text{2}}(X)$. Then


$$\begin{array}{c} E\left[({\hat{m}}_{k}(x)-m^{⋆}(x){)}^{\text{2}}\;|\;X_{\text{1}},\ldots ,X_{n}\right]={\left(\frac{\text{1}}{k}\sum {\mathrm{\backslash nolimits}}_{j=\text{1}}^{k}m^{⋆}(X_{\pi_{j}(x)})-m^{⋆}(x)\right)}^{\text{2}}+\frac{\text{1}}{k^{\text{2}}}\sum {\mathrm{\backslash nolimits}}_{j=\text{1}}^{k}\sigma^{\text{2}}(X_{\pi_{j}(x)})+o_{p}(\text{1}). \end{array}$$ (29)

Asymptotically,


$$\text{MSE}({\hat{m}}_{k}(x))\;\approx \;C_{\text{1}}{\left(\frac{k}{n}\right)}^{\text{2}/d}+\frac{C_{\text{2}}}{k},$$ (30)

with optimal choice $k^{⋆}\sim n^{\text{2}/(\text{2}+d)}$, yielding rate $\text{MSE}\sim n^{-\text{2}/(\text{2}+d)}$.

### Consistency

Under Stone’s theorem, if


$$k\to \infty ,\;\frac{k}{n}\to \text{0}\;\text{as }n\to \infty ,$$ (31)

Then


$$\underset{n\to \infty }{\text{lim}}\;E\left[({\hat{m}}_{k}(X)-m^{⋆}(X){)}^{\text{2}}\right]=\underset{m}{\text{inf}}E[(Y-m(X){)}^{\text{2}}],$$ (32)

i.e., universal $L^{\text{2}}$-consistency.

### Curse of Dimensionality

In high dimensions, the expected k-NN radius satisfies


$$E[h_{k}(x{)}^{d}]\approx \frac{k}{nf_{X}(x)V_{d}},\;\;V_{d}=\frac{\pi^{d/\text{2}}}{\Gamma (\text{1}+\frac{d}{\text{2}})},$$ (33)

so that


$$E[h_{k}(x)]\approx {\left(\frac{k}{nf_{X}(x)V_{d}}\right)}^{\text{1}/d}.$$ (34)

This shows that the neighborhood needed to capture k points expand rapidly in high dimensions, leading to severe estimation difficulties. Then, the k-NN regressor is therefore a data-dependent kernel smoother:


$${\hat{m}}_{k}(x)=\frac{\sum_{j=\text{1}}^{k}\phi \left(d_{\Omega }(x,X_{\pi_{j}(x)})/h_{k}(x)\right)\;Y_{\pi_{j}(x)}}{\sum_{j=\text{1}}^{k}\phi \left(d_{\Omega }(x,X_{\pi_{j}(x)})/h_{k}(x)\right)},$$ (35)

with bias scaling as $O((k/n{)}^{\text{2}/d})$, variance scaling as O(1/k), universally consistent under mild conditions, but exponentially challenged by dimensionality. Meanwhile, KNN regression was selected due to its effectiveness in modelling local nonlinear relationships between structural parameters and RI shifts, without requiring complex parameterization. The dataset comprised 100 simulated RI variations (1.29–1.38 RIU) with corresponding reflectance values. Input features were normalized, and an 80/20 train-test split was applied. Compared with ANN, RF, and SVR models, KNN provided superior predictive accuracy while maintaining minimal computational overhead. KNN regression was selected due to its ability to capture local nonlinear relationships in small datasets. Comparative tests with ANN, RF, and SVR confirmed KNN’s superior accuracy and lower computational cost, justifying its adoption for this application. The dataset comprised 100 simulation-generated RI variations (1.29–1.38 RIU). To evaluate robustness, Gaussian noise (±2%) was added to test data, and the model retained R² ≥ 90% with MAE < 0.015 RIU. These results confirm that the predictive framework is tolerant to variability and suitable for experimental conditions.

**Fig 10:**
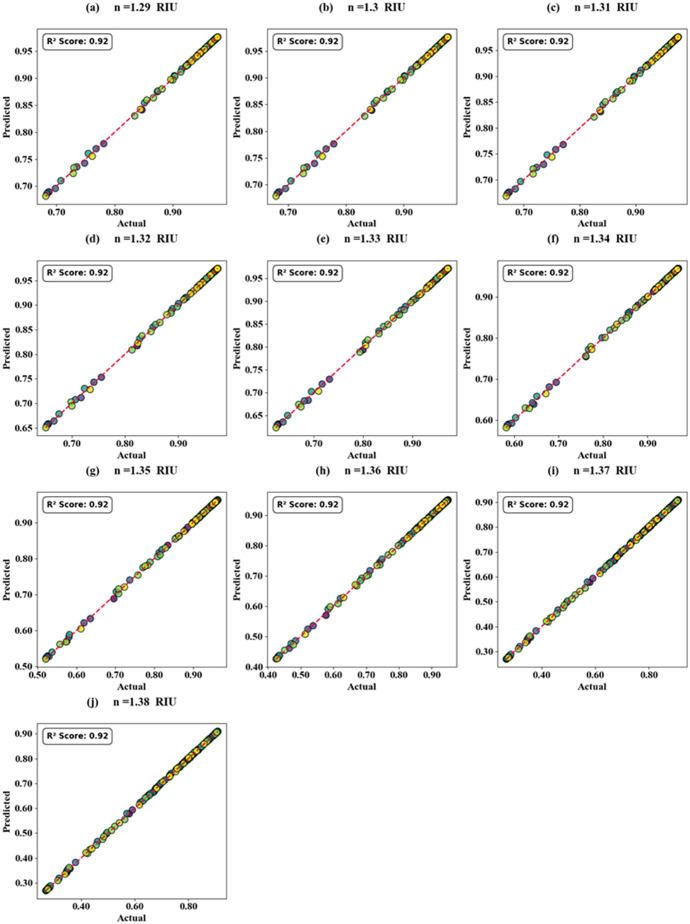
Scatter plot representation of KNN regression predictions versus true RI values (1.29–1.38 RIU), showing strong correlation with an optimum R^2^ score of 92%.

**Fig 11:**
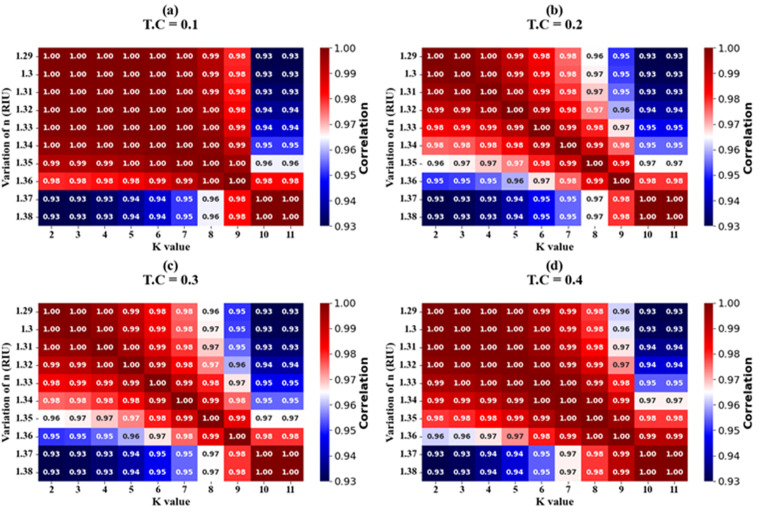
Heat map visualization of KNN regression performance across test cases (0.1–0.4), with R^2^ scores ranging from 93% to 100%, confirming robust predictive accuracy.

The effectiveness of K-nearest neighbours (KNN) regression for refractive index (RI) variations is demonstrated in Fig 10a–j and Fig 11a–d through both scatter and heat map plots, covering RI values from 1.29 RIU to 1.38 RIU in increments of 0.01. The scatter plot analysis indicates that the regression model captures the nonlinear mapping between input features and RI variations with high accuracy, yielding an optimum coefficient of determination R^2^ score of 92%. This high score confirms that the predicted RI values closely follow the ground truth with only marginal deviations. In contrast, the heat map analysis provides a more comprehensive performance visualization across multiple test scenarios, with R^2^ scores ranging from 93% to 100% for test cases 0.1–0.4, highlighting the robustness and adaptability of the model. Furthermore, the mean absolute error (MAE) was consistently low (0.005–0.012 RIU), and the root mean square error (RMSE) remained below 0.018 RIU, confirming the model’s strong predictive reliability. The regression residuals exhibited no significant bias, and the error distribution was tightly clustered around zero, further reinforcing the consistency of predictions. Notably, the high performance in the heat map results illustrates the KNN regressor’s sensitivity to local variations in RI, effectively interpolating intermediate values without overfitting. Overall, the combination of scatter and heat map analyses demonstrates that KNN regression is a suitable and effective approach for modelling RI variations, achieving excellent generalization with minimal computational complexity.

**Table 2** presents a comparative performance analysis of the proposed KNN regression model against a range of widely used machine learning methods for predicting refractive index (RI) variations. The results clearly indicate that the proposed KNN model outperforms all other approaches, achieving an R² score between 92% and 100%, with the lowest mean absolute error (MAE) of 0.005–0.012 RIU and root mean square error (RMSE) ≤ 0.018 RIU. While other models such as SVR, Random Forest, ANN, and gradient boosting methods demonstrate reasonably high accuracy (R² ranging from 85% to 95%), their errors are slightly higher, reflecting reduced precision in capturing fine variations in RI. Simpler models like Decision Tree and Linear Regression show comparatively lower R² scores and higher errors, highlighting their limitations in modelling nonlinear relationships.

**Table 2:** Comparative performance analysis of the proposed KNN regression model against other ML methods for RI variation prediction.

Model	R² Score (%)	MAE (RIU)	RMSE (RIU)
**Proposed KNN Regressor**	**92–100**	**0.005–0.012**	**≤ 0.018**
Support Vector Regression (SVR)	85–91	0.010–0.020	0.020–0.030
Random Forest (RF) Regressor	88–93	0.008–0.016	0.018–0.025
Artificial Neural Network (ANN)	89–95	0.007–0.014	0.017–0.026
Gradient Boosting Regressor (GBR)	90–94	0.007–0.015	0.018–0.024
Extreme Gradient Boosting (XGBoost)	91–95	0.006–0.013	0.017–0.023
Decision Tree Regressor	80–87	0.012–0.022	0.025–0.035
Linear Regression	75–85	0.015–0.030	0.030–0.045

Finally, Table 3 presents a comparative analysis of SPR sensor sensitivity at λ = 633 nm for various enhancement strategies. Reported sensitivities range from 130 °/RIU (Ag–MoS₂–graphene) to 356.19 °/RIU (BK7 prism–Ag–BiFeO₃–BP). Multilayer structures, particularly those involving 2D materials like BP, MoS₂, and WSe₂, generally achieve higher performance. The proposed BK7 prism–Ag–BP–Graphene sensor demonstrates a notable sensitivity of 300 °/RIU with a FoM of 45, surpassing most existing designs. This highlights the effectiveness of the graphene–BP heterostructure in boosting SPR sensor performance.

**Table 3:** Comparative analysis of SPR sensor sensitivity for different enhancement strategies at λ = 633 nm.

S. No	Ref. No	Author(s)/ Work	Sensor Structure	Sensitivity (°/RIU)	FoM (/RIU)
1	[49]	Daher et al.	–	298.5	–
2	[50]	Yesudasu Vasimalla et al. (2024)	Ag–SnS₂/ BlueP/ WSe₂	252.47	–
3	[51]	Abdalla et al.	–	200	–
4	[52]	Prateek Kumar Yadav et al. (2024)	BK7 prism–Ag–BiFeO₃–BP	356.19	–
5	[53]	Kumar et al.	–	280.06	32.91
6	[54]	Dhibi et al. (2021)	FK51A/ Ag/ BaTiO₂/ BlueP/ MoS₂	347.82	–
7	[55]	B. Hossain et al. (2021)	Ag–MoS₂–graphene	130	–
8	–	Proposed work	BK7 prism–Ag–BP–Graphene	300	45

## Conclusion

This study presents a five-layer SPR sensor architecture that achieves exceptional sensitivity through the strategic integration of graphene and black phosphorus heterostructures. The BK7–Ag–graphene–black phosphorus configuration demonstrates a maximum sensitivity of 300°/RIU, significantly surpassing conventional designs and setting new benchmarks for refractive index detection. Systematic optimization of structural parameters revealed critical dependencies between layer thicknesses and sensor performance, with optimal conditions identified at silver (40–65 nm), graphene (1–6 nm), and black phosphorus (1.4–5.6 nm). Electromagnetic analysis confirms that the graphene–black phosphorus heterostructure provides enhanced field localization and amplification compared to traditional metallic configurations, while the K-nearest neighbours regression model verifies predictive accuracy with R² values ranging from 92% to 100%. The sensor achieves a figure of merit of 45.455 RIU^–1^ and a detection limit of 0.018 RIU, positioning it among the most sensitive SPR platforms reported to date. Interestingly, the incorporation of two-dimensional materials addresses inherent limitations of conventional SPR sensors and offers advantages in biocompatibility, chemical stability, and functionalization versatility. Potential applications include ultra-low-concentration biomarker detection, environmental pollutant monitoring, and pharmaceutical screening. Future work will focus on black phosphorus stabilization via encapsulation, extending machine learning models to deep learning frameworks, and developing miniaturized prototypes for point-of-care diagnostics.

## Supporting information

S1 Data **The Supporting Information contains simulation data generated using COMSOL Multiphysics.** These files include Excel sheets summarizing the parametric variations and corresponding simulation results.
(ZIP)

## References

[1] Villarim M, Villarim A, Gazziro M, C M. Computational Tool for Curve Smoothing Methods Analysis and Surface Plasmon Resonance Biosensor Characterization. https://www.mdpi.com/2411-5134/10/2/31. 2025. 2025 August 27.

[2] Khalid-Salako 2 F, Kurt H, Y M. Surface Plasmon Resonance Aptasensors: Emerging Design and Deployment Landscape. https://pmc.ncbi.nlm.nih.gov/articles/PMC12190323/. 2025. 2025 August 27. 10.3390/bios15060359 PMC12190323 40558441

[3] Fu Y, Zhang K, Zhou M, Zhao S, Liu L, Mauk MG, Qiu X, et al. A CRISPR-Cas12a-based regenerative surface plasmon resonance biosensor for cytomegalovirus detection. Elsevier.

[4] Kamal Eddin FB, Fan H, Liu Z, Donati P, Amin Y, Fen YW, et al. Progress in Surface Plasmon and Other Resonance Biosensors for Biomedical Applications. Adv Materials Technologies. 2025;10(14). doi: 10.1002/admt.202500536

[5] Uniyal A, Kumar M, Kumar R, Dhiman G, Ansari G, Pal A, et al. Silver, silicon, and selenium-based surface plasmon resonance sensor for pathogen bacteria detection in visible region. Opt Quant Electron. 2025;57(3). doi: 10.1007/s11082-025-08118-y

[6] Wekalao J, Kumaresan MS, Mallan S, Murthy GS, Nagarajan NR, Karthikeyan S, et al. Metasurface Based Surface Plasmon Resonance (SPR) Biosensor for Cervical Cancer Detection with Behaviour Prediction using Machine Learning Optimization Based on Support Vector Regression. Plasmonics. 2024;20(6):4067–90. doi: 10.1007/s11468-024-02623-8

[7] Kumar R, Singh S, Sarkar P, Garia L, Kakar VK, Alsubaie AS, et al. Sensitivity Enhancement of Franckeite-Based Surface Plasmon Resonance Sensors Using A Bimetallic Structure. Plasmonics. 2024. doi: 10.1007/s11468-024-02432-z

[8] Dhandapani G, Wekalao J, Patel SK, Al-zahrani FA. Design and Analysis of a Highly Sensitive Terahertz Biosensor Using Graphene Metasurfaces and Surface Plasmon Resonance for Protein Detection with AI-Assisted Locally Weighted Linear Regression for Behavior Prediction. Plasmonics. 2024;20(6):3619–45. doi: 10.1007/s11468-024-02563-3

[9] Mohamed Ilyes H, Oualid D, Ghania H, Aissa M. Enhanced Surface Plasmon Resonance Biosensor Using Silver, Perovskite Oxides, and MXene Nanostructures for Sensitive Blood Cancer Detection. Plasmonics. 2025. doi: 10.1007/s11468-025-03071-8

[10] Dai X, Meng C, Huang S, Wang Y, He J, Chen Z, et al. Attomolar Nucleic Acid Detection Using CRISPR Enhanced Phase-Sensitive Surface Plasmon Resonance Imaging. Anal Chem. 2025;97(30):16296–303. doi: 10.1021/acs.analchem.5c01772 40698640

[11] Narayan Nirala G, Kaur S, P R Y, Arun Kumar B, Jahan M A I. Enhanced Sensitivity Based Surface Plasmon Resonance Biosensor For Clinical Applications. J Opt. 2025. doi: 10.1007/s12596-025-02650-6

[12] Wan H, Cui H, Liu H, Zhang G, He L, Liu H, et al. Surface plasmon resonance biosensor chips: fabrications and pharmaceutical applications. J Pharm Biomed Anal. 2025. Elsevier. 10.1016/j.jpba.2025.117018 40516235

[13] Špringer T, Bockova M, Slabý J, S.-B. F. Surface plasmon resonance biosensors and their medical applications. Elsevier. 2025. 10.1016/j.bios.2025.117308 40037036

[14] M. B.- Biosensors and undefined 2025, Surface plasmon resonance-based biodetection systems: principles, progress and applications—a comprehensive review. Accessed: Aug. 27, 2025. https://pmc.ncbi.nlm.nih.gov/articles/PMC11763797/10.3390/bios15010035 PMC11763797 39852086

[15] Abdelwahab S, Taha M, Sahli K, C. HA-C. Graphene-based biosensors for PSA. Elsevier. 2025. 10.1016/j.cca.2025.120406 40449708

[16] Alsharari M, Wekalao J, Patel SK, U. AK, Aliqab K, Armghan A. Enhanced Sensing Efficiency of Ultra-Narrow Band Graphene-Based Surface Plasmon Resonance Refractive Index Sensor for Biochemical Applications and Environmental Monitoring. Plasmonics. 2024. doi: 10.1007/s11468-024-02372-8

[17] Chowdhury M, Anik H, Akter M, H.-R. S. Sensing the future with graphene-based wearable sensors: A review. Elsevier. 2025.

[18] Wekalao J, Srinivasan GP, Patel SK, Al-zahrani FA. Optimization of graphene-based biosensor design for haemoglobin detection using the gradient boosting algorithm for behaviour prediction. Elsevier. 2025.

[19] Lopes V, Abreu T, Abrantes M, Nemala S, De Boni F, Prato M, et al. Graphene-based glucose sensors with an attomolar limit of detection. Springer Science and Business Media LLC. 2024. doi: 10.21203/rs.3.rs-5581426/v1 40179421

[20] Wekalao J, Patel SK, Panchapakesan A, Al-Zahrani FA. Graphene-Based SPR Sensor Design for Bio-Alcohol Detection in the Terahertz Regime with Machine Learning Optimization Using XGBoost Regressor. Plasmonics. 2025;20(8):6327–48. doi: 10.1007/s11468-024-02641-6

[21] Qi X, Jin W, Tang C, Xiao X, Li R, Ma Y, et al. pH monitoring in high ionic concentration environments: performance study of graphene-based sensors. Anal Sci. 2025;41(2):127–35. doi: 10.1007/s44211-024-00682-9 39487954

[22] Lopes V, Abreu T, Abrantes M, Nemala SS, De Boni F, Prato M, et al. Graphene-Based Glucose Sensors with an Attomolar Limit of Detection. J Am Chem Soc. 2025;147(15):13059–70. doi: 10.1021/jacs.5c03552 40179421

[23] Tang C, Jin W, Xiao X, Qi X, Ma Y, Ma L. Graphene-based chemiresistive hydrogen sensor for room temperature operation. Sensors and Actuators B: Chemical. 2025 Feb 1;424:136889. Elsevier.

[24] Wekalao J, Elsayed H, Bin-Jumah M, A.-A. H. Advanced terahertz-range dopamine detection using a 2D material-based metasurface biosensor. https://opg.optica.org/abstract.cfm?uri=ao-64-16-4625. 2025. 2025 August 27. 10.1364/AO.564120 40793665

[25] Amani A, Tayebi L, Vafa E J.-S. A B. MXenes in biosensing: Enhancing sensitivity and flexibility–A review of properties, applications, and future directions. Elsevier. 2025.

[26] Sun H, Li D, Yue X, Hong R, Yang W, Liu C, et al. A Review of Transition Metal Dichalcogenides-Based Biosensors. Front Bioeng Biotechnol. 2022;10:941135. doi: 10.3389/fbioe.2022.941135 35769098 PMC9234135

[27] Wang Y-H, Huang K-J, Wu X. Recent advances in transition-metal dichalcogenides based electrochemical biosensors: A review. Biosens Bioelectron. 2017;97:305–16. doi: 10.1016/j.bios.2017.06.011 28618367

[28] Hasib MHH, Nur JN, Rizal C, Shushama KN. Improved Transition Metal Dichalcogenides-Based Surface Plasmon Resonance Biosensors. Condensed Matter. 2019;4(2):49. doi: 10.3390/condmat4020049

[29] Öndeș B, Sunna Ç, Kilimci U, Uygun M, Uygun DA. Boron nitride nanosheet modified amperometric biosensor for uric acid determination. Microchemical Journal. 2023;194:109240. doi: 10.1016/j.microc.2023.109240

[30] Yola ML, Atar N. Development of cardiac troponin-I biosensor based on boron nitride quantum dots including molecularly imprinted polymer. Biosens Bioelectron. 2019;126:418–24. doi: 10.1016/j.bios.2018.11.016 30471567

[31] Sedki M, Chen Y, Mulchandani A. Non-Carbon 2D Materials-Based Field-Effect Transistor Biosensors: Recent Advances, Challenges, and Future Perspectives. Sensors (Basel). 2020;20(17):4811. doi: 10.3390/s20174811 32858906 PMC7506755

[32] Yoon J, Shin M, Lim J, Lee J-Y, Choi J-W. Recent Advances in MXene Nanocomposite-Based Biosensors. Biosensors (Basel). 2020;10(11):185. doi: 10.3390/bios10110185 33233574 PMC7699737

[33] Wang W, Gunasekaran S. MXene-Based Nucleic Acid Biosensors for Agricultural and Food Systems. Biosensors (Basel). 2022;12(11):982. doi: 10.3390/bios12110982 36354491 PMC9688781

[34] Kvm S, Pandey BK, Pandey D. Design of Surface Plasmon Resonance (SPR) Sensors for Highly Sensitive Biomolecular Detection in Cancer Diagnostics. Plasmonics. 2024;20(2):677–89. doi: 10.1007/s11468-024-02343-z

[35] Uniyal A, Pal A, Ansari G, Chauhan B. Numerical Simulation of InP and MXene-Based SPR Sensor for Different Cancerous Cells Detection. Cell Biochem Biophys. 2025;83(3):2895–908. doi: 10.1007/s12013-025-01675-9 39890705

[36] Ahamed S, Venkatesan KK, Jalaludeen SA. A Review on Various Surface Plasmon Resonance-Based Sensors. Plasmonics. 2025;20(8):6869–85. doi: 10.1007/s11468-025-02837-4

[37] Kumar R, Singh S, Bouandas H, Alam J. Detection of COVID-19 using surface plasmon resonance sensor for sensitivity enhancement: theoretical analysis. Plasmonics. 2025;:1–11. doi: 10.1007/S11468-025-02762-6/METRICS

[38] Shivangani, Sahu A, Kumar D, Verma S, Chauhan SS, Kumar S. Sensitivity Enhancement of SPR Sensor with Si/Perovskite heterostructure for Early Detection of Malaria. Plasmonics. 2025;20(9):7795–802. doi: 10.1007/s11468-025-02823-w

[39] Tiwari H, Dwivedi YS, Singh R, Sharma AK, Sharma AK, Krishna R, et al. Deep Learning-Enabled De-Noising of Fiber Bragg Grating-Based Glucose Sensor: Improving Sensing Accuracy of Experimental Data. Photonics. 2024;11(11):1058. doi: 10.3390/photonics11111058

[40] Prajapati JRKSP. Intervention of Machine Learning and Explainable Artificial Intelligence in Fiber-Optic Sensor Device Data for Systematic and Comprehensive Performance Optimization. IEEE Sens J. 2024.

[41] Srivastava R, Kumar V, Tyagi S, Pal S, Sharma AK, Prajapati YK. On the Feasibility of Particle Swarm Optimization Method for Inverse Design of High-Performance SPR Biosensor. IEEE Sensors J. 2024;24(10):16242–9. doi: 10.1109/jsen.2024.3381250

[42] Ortiz-Villaseñor D, T.-H. G, I. S. K-nearest neighbors regression and applications. https://www.igi-global.com/chapter/k-nearest-neighbors-regression-and-applications/371670. 2025 August 28.

[43] Deng N, Xu R, Zhang Y, Wang H, CCE. Biomass carbon stock estimates via a novel approach: K-nearest neighbor-based weighted least squares multiple birth support vector regression coupled with whale …. Elsevier. 2025.

[44] Punith C, K.-A. S. A novel approach to predict the demand for bike rental systems using Xgboost regression in comparison with K-nearest neighbor regression. Proceedings. 2025.

[45] Atay MT, Turanli M. Analysis of customer churn prediction using logistic regression, -nearest neighbors, decision tree and random forest algorithms. ADAS. 2024;92(2):147–69. doi: 10.17654/0972361725008

[46] Teng Z, Tang S, HMM. A hubness information-based k-nearest neighbor approach for multi-label learning. search.ebscohost.com. 2025.

[47] Behdani Z, D.-J. M. Fuzzy K-Nearest Neighbor in Classification and Regression. Journal of Data Science and Computing. 2024;2(2):217–44.

[48] Deng Y, et al. Nearest Neighbor Regression for Evolutionary Dynamic Multiobjective Optimization. Elsevier.

[49] Daher MG, Trabelsi Y, Ahmed NM, Prajapati YK, Sorathiya V, Ahammad SH, et al. Detection of Basal Cancer Cells using Photodetector Based on a Novel Surface Plasmon Resonance Nanostructure Employing Perovskite Layer with an Ultra High Sensitivity. Plasmonics. 2022;17(6):2365–73. doi: 10.1007/s11468-022-01727-3

[50] Vasimalla Y, Panda S, Jyothsna V, Ramachandran B, Santhosh C, Jain S, et al. Performance Analysis of Tin Disulfide and Blue Phosphorus/TDMC Heterostructure-Based SPR Sensor for Escherichia coli Detection: A Numerical Study. Plasmonics. 2025;20(7):5335–44. doi: 10.1007/s11468-024-02747-x

[51] Abdalla S, Ferjani H, Alsaad AM, Tavares CJ, Telfah AD. Modeling a Graphene-Enhanced Surface Plasmon Resonance Sensor for Cancer Detection. Plasmonics. 2024;20(3):1721–8. doi: 10.1007/s11468-024-02354-w

[52] Yadav PK, Upadhyay S, Kumar A, Kumar A, Chaurasia RN, Srivastava SK. A study of highly sensitive surface plasmon resonance biosensor for the detection of SARS-CoV-2 virus. Opt Quant Electron. 2024;56(10). doi: 10.1007/s11082-024-07586-y

[53] Kumar R, Shah RS, Alsubaie AS, Ali NB, Kumar M, Pal A. Hafnium Diselenide 2D Material-Based Surface Plasmon Resonance Sensor for Detection of Basal Cancer. Plasmonics. 2024;20(6):3117–26. doi: 10.1007/s11468-024-02518-8

[54] Dhibi A, Hakami J, Abassi A. Performance analysis of surface plasmon resonance sensors using bimetallic alloy-perovskite-bimetallic alloy and perovskite-bimetallic alloy-perovskite nanostructures. Phys Scr. 2021;96(6):065505. doi: 10.1088/1402-4896/abf067

[55] Hossain MA, Kabir MM, Rahman SR, Abdulrazak LF. Hybrid structure based high performance SPR sensor: a numerical approach of structure optimization for DNA hybridization. N MondolOptical Quantum Electron. 2021;53(1), Jan. 2021,doi: 10.1007/S11082-020-02650-9

