# Expression of Concern: Enhanced surface plasmon resonance biosensor with graphene-black phosphorus heterostructure for ultra-high sensitivity refractive index detection with machine learning for behaviour prediction

**DOI:** 10.1371/journal.pone.0345133

**Published:** 2026-03-19

**Authors:** The PLOS One Editors

After this article [1] was published, the *PLOS One* Editors identified concerns about compliance with PLOS policies on Authorship and Artificial Intelligence Tools and Technologies.

In light of these concerns, the *PLOS One* Editors issue this Expression of Concern. Readers are advised to interpret the article [1] with caution.
