# Supplementary figures and images for: Enhanced surface plasmon resonance biosensor with graphene-black phosphorus heterostructure for ultra-high sensitivity refractive index detection with machine learning for behaviour prediction
Source: PLoS One. 2025 Nov 7;20(11):e0332356. doi: 10.1371/journal.pone.0332356 (PMC12594348; doi:10.1371/journal.pone.0332356)

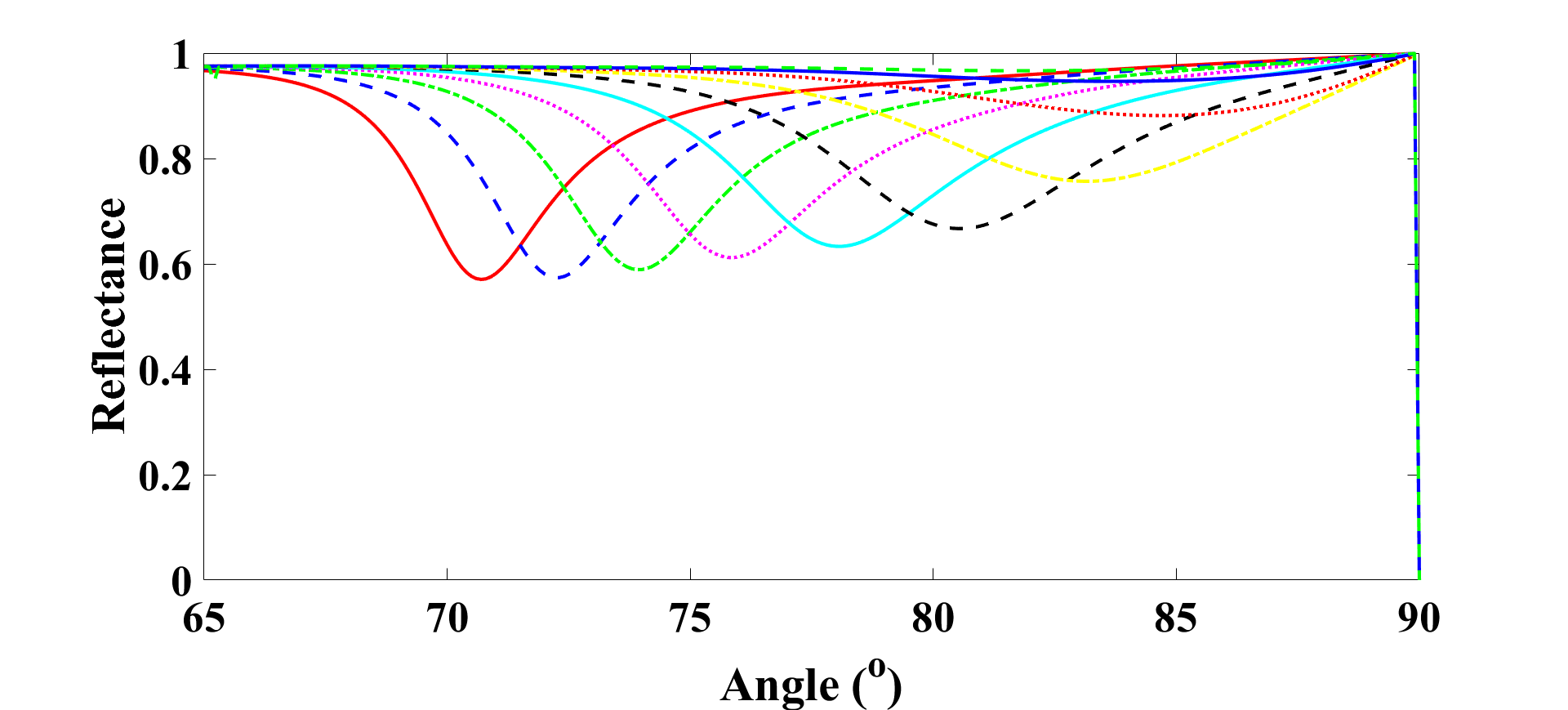

Supplement: S1 Data — The Supporting Information contains simulation data generated using COMSOL Multiphysics. These files include Excel sheets summarizing the parametric variations and corresponding simulation results. (ZIP) [file pone.0332356.s001.zip › ag/untitled.png]

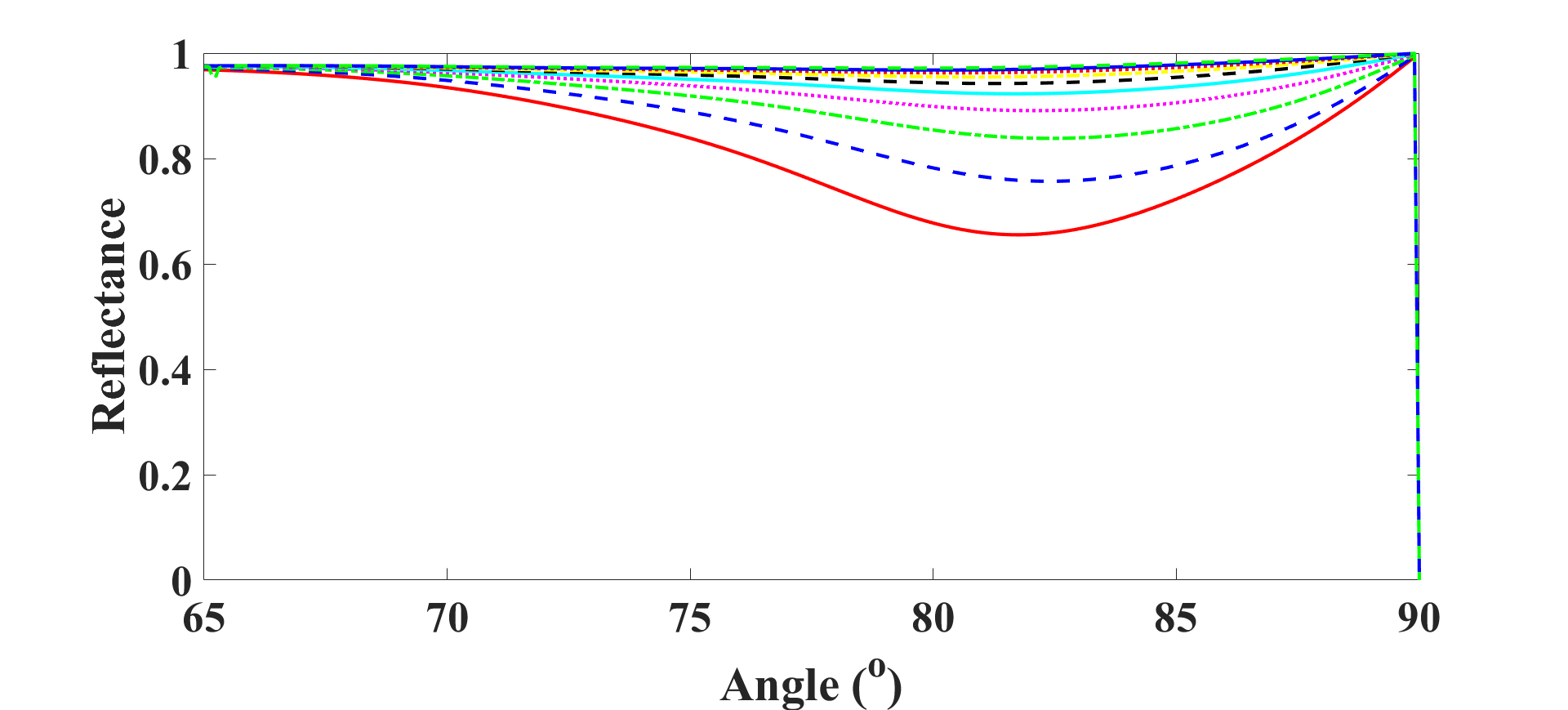

Supplement: S1 Data — The Supporting Information contains simulation data generated using COMSOL Multiphysics. These files include Excel sheets summarizing the parametric variations and corresponding simulation results. (ZIP) [file pone.0332356.s001.zip › graphene/untitled.png]

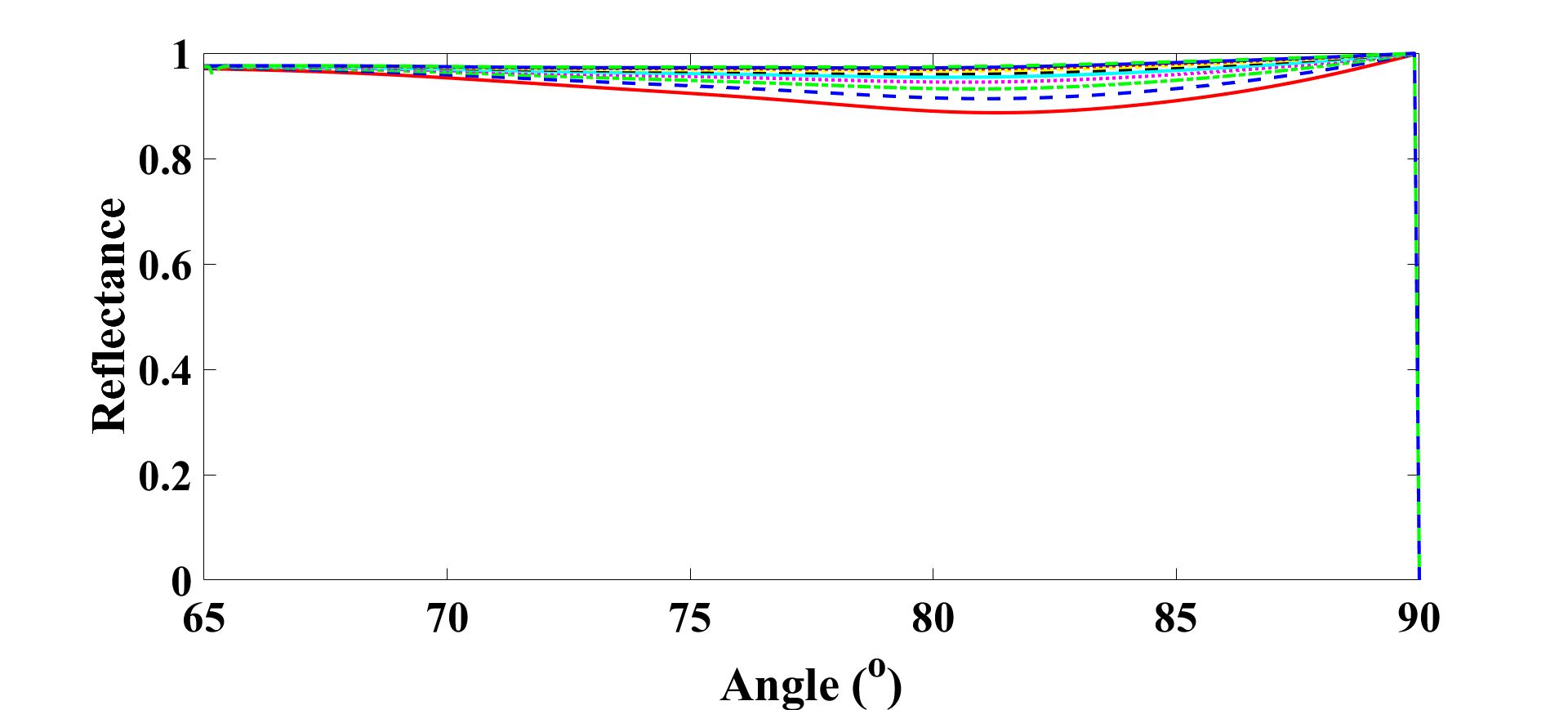

Supplement: S1 Data — The Supporting Information contains simulation data generated using COMSOL Multiphysics. These files include Excel sheets summarizing the parametric variations and corresponding simulation results. (ZIP) [file pone.0332356.s001.zip › WS2/untitled.png]
